# Pyruvate kinase M2 and the mitochondrial ATPase Inhibitory Factor 1 provide novel biomarkers of dermatomyositis: a metabolic link to oncogenesis

**DOI:** 10.1186/s12967-017-1136-5

**Published:** 2017-02-10

**Authors:** Fulvio Santacatterina, María Sánchez-Aragó, Marc Catalán-García, Glòria Garrabou, Cristina Nuñez de Arenas, Josep M. Grau, Francesc Cardellach, José M. Cuezva

**Affiliations:** 1 Departamento de Biología Molecular, Centro de Biología Molecular Severo, Ochoa, CSIC-UAM, Universidad Autónoma de Madrid, c/Nicolás Cabrera 1, 28049 Madrid, Spain; 2 Centro de Investigación Biomédica en Red de Enfermedades Raras (CIBERER), Madrid, Spain; 3 Instituto de Investigación Hospital 12 de Octubre, ISCIII, Madrid, Spain; 4 Muscle Research and Mitochondrial Function Laboratory, CELLEX-IDIBAPS, Faculty of Medicine-University of Barcelona, Internal Medicine Department-Hospital Clinic of Barcelona, Barcelona, Spain

**Keywords:** Biomarkers, Inflammatory myopathies, Dermatomyositis, Pyruvate kinase M2, ATPase Inhibitory Factor 1, Energy metabolism, Mitochondria

## Abstract

**Background:**

Metabolic alterations play a role in the development of inflammatory myopathies (IMs). Herein, we have investigated through a multiplex assay whether proteins of energy metabolism could provide biomarkers of IMs.

**Methods:**

A cohort of thirty-two muscle biopsies and forty plasma samples comprising polymyositis (PM), dermatomyositis (DM) and sporadic inclusion body myositis (sIBM) and control donors was interrogated with monoclonal antibodies against proteins of energy metabolism using reverse phase protein microarrays (RPPA).

**Results:**

When compared to controls the expression of the proteins is not significantly affected in the muscle of PM patients. However, the expression of β-actin is significantly increased in DM and sIBM in consistence with muscle and fiber regeneration. Concurrently, the expression of some proteins involved in glucose metabolism displayed a significant reduction in muscle of sIBM suggesting a repression of glycolytic metabolism in these patients. In contrasts to these findings, the expression of the glycolytic pyruvate kinase isoform M2 (PKM2) and of the mitochondrial ATPase Inhibitor Factor 1 (IF1) and Hsp60 were significantly augmented in DM when compared to other IMs in accordance with a metabolic shift prone to cancer development. PKM2 alone or in combination with other biomarkers allowed the discrimination of control and IMs with very high (>95%) sensitivity and specificity. Unfortunately, plasma levels of PKM2 were not significantly altered in DM patients to recommend its use as a non-invasive biomarker of the disease.

**Conclusions:**

Expression of proteins of energy metabolism in muscle enabled discrimination of patients with IMs. RPPA identified the glycolysis promoting PKM2 and IF1 proteins as specific biomarkers of dermatomyositis, providing a biochemical link of this IM with oncogenesis.

**Electronic supplementary material:**

The online version of this article (doi:10.1186/s12967-017-1136-5) contains supplementary material, which is available to authorized users.

## Background

Inflammatory myopathies (IMs) is a group of heterogeneous diseases characterized by muscle weakness and inflammatory infiltrates within the skeletal muscle. Despite presenting unknown etiology, inflammatory and bioenergetic disturbances have been argued in most of cases. Due to their similar clinical presentation, polymyositis (PM), dermatomyositis (DM) and sporadic inclusion-body myositis (sIBM) are the three major groups ascribed to IMs [1]. A fourth and fifth subtypes termed necrotizing auto-immune myositis and overlap myositis are also being recognized within the group of IMs [2]. IMs are considered rare diseases due to their low incidence, about of 2.1 to 7.7 new cases per every million inhabitants/year. sIBM is the most common acquired myopathy in patients above 50 years, with some geographical differences [3–5]. There are few biomarkers that could help the diagnosis and management of patients affected by IMs, in particular myositis-associated or specific antibodies. The elevated serum activity of creatine kinase, lactate dehydrogenase and aldolase are currently used as activity indicators of all subtypes of IMs [2].

Reverse phase protein microarrays (RPPA) is a high-throughput quantitative technique adequate for multiplexed analysis of protein expression in minute amounts of sample in a large variety of biological specimens [6, 7]. Over the last decade, RPPA technique has provided a precious tool in the discovery of biomarkers of disease which might become indispensable in the progress of diagnostic, prognostic and therapeutic fields. The Achilles heel for the development of a reliable RPPA platform, is the availability of high-affinity and specific antibodies against the proteins investigated [8, 9]. Herein, we have studied the putative relevance of proteins of energy metabolism as diagnostic biomarkers in IMs using RPPA. To this aim, we have studied the expression of enzymes of glucose metabolism and of oxidative phosphorylation in a cohort of thirty-two muscle biopsies including samples from control and PM, DM and sIBM affected patients using validated monoclonal antibodies. The final purpose of the study is to translate the “signature” of energy metabolism to bed-side application of patients affected with IMs.

## Methods

### Patients and protein extraction

A cohort of thirty-two muscle biopsies from a deltoid or quadriceps muscle was collected. The muscle biopsy was immediately frozen in liquid N_2_ cooled isopenthane and stored at −80 °C until histological sectioning for diagnostic procedures. In brief, 8–10 microns cryotome sections were obtained and processed for histopathological and molecular and clinical diagnosis by the same expert pathologist (JMG). The final diagnosis of the cohort was: 4 PM, 13 DM, 9 sIBM and 6 healthy controls. In all the cases the biopsies were obtained for diagnostic purposes, and all the patients signed an informed consent before the procedure for further used of their samples in research. In addition, forty plasma samples of control donors (n = 10) and patients affected with IMs including PM (n = 5), DM (n = 9) and sIBM (n = 16) were also collected. The samples were obtained from leftover biological material from diagnostic procedures with informed consent following the Declaration of Helsinki and coded for anonymity to protect patient confidentiality. The Institutional Review Board approved the project. For protein extraction from muscle biopsies, the samples were homogenized in T-PER Tissue Protein Extraction Reagent (ThermoScientific, Inc.) containing protease inhibitors (Roche) in a 1:5 (w/v) ratio, and further freeze–thawed three times in liquid nitrogen [10]. The protein concentration was determined with the Bradford reagent (Bio-Rad, Inc) using BSA as standard.

### Protein electrophoresis and western blotting

Protein samples from muscle biopsies were fractionated on SDS–9% PAGE and blotted with anti-β-F1-ATPase (1:1000), anti-Hsp60 (1:1000), anti-GAPDH (1:1000) and anti-PKM2 (1:1000) from [11], anti-IF1 (1:500) from [12], anti-LDH-A (1:1000), anti-GPD1 (1:1000) from [13], anti-PYGM (Abcam, ab88078; 1:1000), anti-PKM1 (Abcam, ab6191-5; 1:1000), anti phospho-PKM2 (Tyr105) (Cell Signaling, #3827; 1:1000), anti-PDHE1α (Invitrogene, 459400; 1:500), anti-phospho-PDHE1α (Ser293) (Abcam, ab92696; 1:1000), anti-PDK1 (Abcam, ab207450; 1:1000) and anti-β-actin (Sigma-Aldrich, A1978; 1:1000). Peroxidase-conjugated anti-mouse IgGs (Nordic Immunology; 1:3000) were used as secondary antibodies. The blots were revealed using the ECL^®^ reagent (Amersham Pharmacia Biotech). The intensity of the bands was quantified using a Kodak DC120 digital camera and the Kodak 1D Analysis Software.

### Printing and processing of reverse phase protein microarrays

Samples from muscle biopsies were diluted in PBS to a final protein concentration of 0.75 μg/μl before printing. Serially diluted protein extracts (0–1 μg/μl) derived from HCT116 colocarcinoma cells were also prepared to assess printing quality and the linear response of protein recognition by the antibodies used. A standard curve of BSA (0–1 μg/μl) and mouse IgGs (0–1 ng/ml) were also prepared for printing as internal negative and positive controls, respectively. Plasma samples were diluted 1:20 (v/v) in PBS before printing. A Standard curve (0–1 ng/ml) of the PKM2 recombinant protein (r-PKM2) was printed to correlate the mean fluorescent intensity of the samples to the quantity of the protein. Approximately, 1 nl volume of each sample was spotted in triplicate onto nitrocellulose-coated glass slides (FAST Slides, Scheleicher & Schuell BioScience, Inc.) using a BioOdyssey Calligrapher MiniArrayer printer (Bio-Rad Laboratories, Inc.) equipped with a solid pin (MCP310S) at constant humidity (RH 45%) and temperature (16 °C).

After printing, arrays were allowed to dry and further blocked in PBS-T containing 5% skimmed milk. An additional blocking step with goat anti-human whole serum IgGs (Sigma-Aldrich, I1011; 1:100) was necessary to prevent the cross reaction of the secondary antibody with plasma IgGs. After, the arrays were incubated overnight at 4 °C with the indicated concentrations of the following primary monoclonal antibodies: anti-β-F1-ATPase (1:150), anti-Hsp60 (1:150), anti-GAPDH (1:250) and anti-PKM2 (1:150), anti-IF1 (1:150), anti-LDH-A (1:200), anti-GPD1 (1:1000), anti-PYGM (1:200) and anti-β-actin (1:1000). After incubation, the arrays were washed with PBS-T and further incubated with a donkey anti-mouse secondary antibody conjugated with alexa-555 (Invitrogen, Madrid, Spain) or, in the case of arrays of plasma samples, incubated with goat anti-mouse highly cross-adsorbed antibody conjugated with CF™647 (Sigma-Aldrich, SAB4600183; 1:500). To evaluate the unspecific binding of the secondary antibody to non-masked human IgGs in plasma samples, a pad was incubated directly with the secondary antibody as negative control of the RPPA analysis. Spotted samples in one of the pads were fixed with XFCF buffer (10% acetic acid, 30% ethanol) for 1 h, stained with 0.0001% Fast Green FCF (Sigma-Aldrich, F7252) in XFCF for 5 min and washed 5 times with XFCF in order to quantify the total protein amount of each spot. Microarrays were scanned using a Typhoon 9410 scanner (GE Healthcare, Inc.). The mean fluorescent intensity of the spots was quantified using GenePix^®^ Pro 7 software system and converted into arbitrary units of expressed protein/ng of protein in the sample using the expression obtained in the standard curve of the HCT116 cell line and normalized to the protein amount in the sample obtained from the FCF stained pad. Representative technical variances of the PKM2 arrays, calculated by the squared coefficient of variation (SCV  =  σ*100/|x|), were 2.7  ±  0.4 for muscle samples and 3.2 ± 0.4 for plasma samples.

### Indirect ELISA

Plasma samples (50 μl) from IBM (n = 8), DM (n = 9) patients and a control group (n = 8) were absorbed in F96 IMMUNO PLATE (NUNC, 442404) for 1 h at 37 °C. A standard curve (0–4 μg/ml) of the recombinant PKM2 (r-PKM2) [11] was also adsorbed. After blocking and extensive washing, 50 μl of a solution containing mouse anti-PKM2 (1:150) were added to each well and incubated at 37 °C for 1 h. Wells were washed with PBS-T, and after 50 μl of horseradish peroxidase-labeled goat anti-mouse IgG (1:1000) (Bio-Rad Laboratories, 172-1011) were added. Color development was achieved by addition of 200 μl of the peroxidase substrate solution (Bio-Rad Laboratories, Inc., 172-1064). Reaction was stopped by addition of 100 μl/well of 2% oxalic acid. The absorbance at 415 nm was measured with a FLUOstar OMEGA Microplate Reader (BMG LABTECH).

### Immunofluorescence microscopy

Formalin fixed cryostat tissue sections were used to analyze the subcellular localization of PKM2 in muscle of control and DM patients. The primary anti-PKM2 antibody was used at a 1:200 dilution. Slides were incubated for 2 h in the dark with anti-mouse IgGs conjugated to Alexa Fluor^®^ 488. Nuclei were counter stained with DAPI (diamidino-2-fenilindol) reagent. Cellular fluorescence was analyzed by confocal microscopy in a Nikon A1R + microscope.

### Statistical analysis

Distribution of molecular markers was studied by using a two-tailed Student’s t test. Analysis of variance (ANOVA) with post hoc Dunnett’s test was used for multiple comparisons to the control. Inter-individual variation of the expression of protein levels in muscle biopsies were shown in box plots graphs, using the PASW statistics 18 software package. For the expression profiles of metabolic markers data were reformatted by calculating the log2 of the expression level in each sample relative to the mean expression level in normal samples. We used the Cluster Program from “Expression Profiler Clustering home page” at http://ep.ebi.ac.uk/EP/EPCLUST by the Average Linkage (Weighted Pair Group Method Average WPGMA) clustering based on the Euclidean distance function as proximity measure. Nonparametric receiver operating characteristic (ROC) curves were generated to plot the sensitivity of the assay against the false-positive rate (1-specificity). The area under the curve (AUC) was calculated to illustrate the sensitivity and specificity of the biomarkers. The results shown are mean ± S.E.M. A p < 0.05 was considered statistically significant.

## Results

**Fig. 1:**
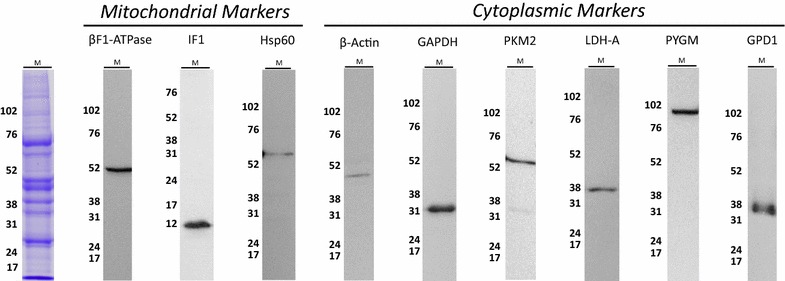
Validation of the antibodies used in RPPA. Tissue extracts (40 µg) derived from human muscle (M) were fractionated on SDS-PAGE gels, blotted against the indicated antibodies and processed for western blotting. Only antibodies that recognize a single protein band of the expected molecular mass were used in the study. The migration of molecular mass markers is indicated to the *left*

To explore the potential applicability of a signature of metabolism in the diagnosis of IMs we selected for study representative proteins from glycogenolysis (Glycogen phosphorylase, PYGM), glycolysis (glyceraldehyde-3-phosphate dehydrogenase, GAPDH, pyruvate kinase M2, PKM2 and lactate dehydrogenase A, LDHA), oxidative phosphorylation (β-subunit of the H^+^-ATP synthase, β-F1-ATPase and ATPase Inhibitory Factor 1, IF1), and electron shuttling of glycolytic NADH to mitochondria (glycerol-phosphate dehydrogenase 1, GPD1). In addition, β-actin and heat shock protein 60 kDa (Hsp60) were also studied as structural markers of the cell and mitochondria, respectively. The major limitation of quantitative RPPA is the availability of specific monoclonal antibodies (mAbs) against the proteins being studied. Figure 1 shows that the mAbs used in this study only recognized a single protein band at the expected molecular weight in human muscle extracts validating their utilization in RPPA techniques [11–13].

**Fig. 2:**
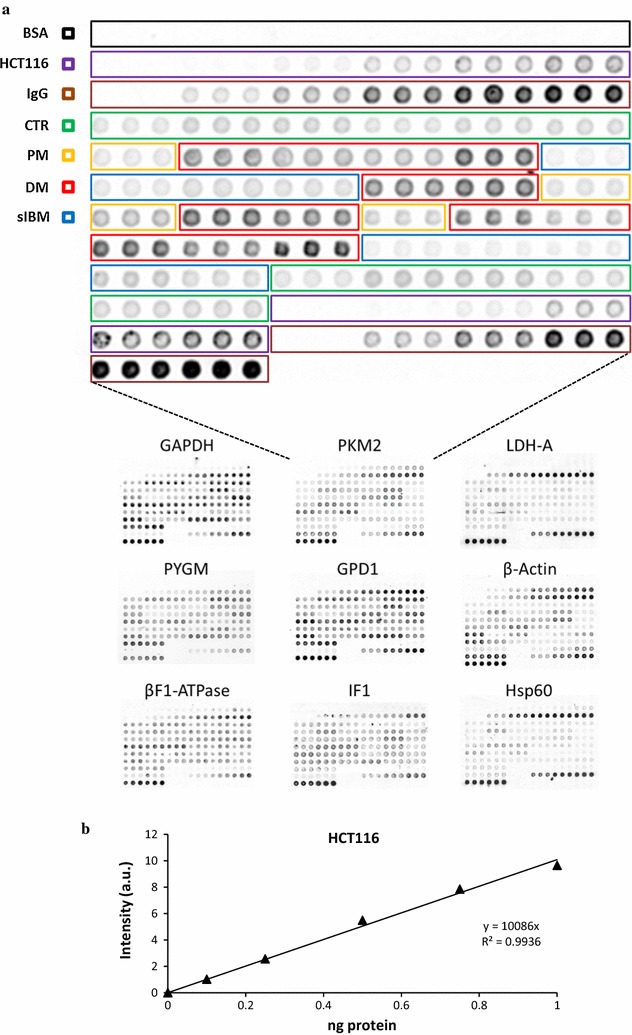
Printing of RPPA. **a** The scheme of printing of RPPA processed for PKM2 is shown magnified for details. One nl samples were spotted in triplicate. *Black boxed* negative controls of BSA; *Magenta boxed* standard curves of HCT116 cells; *Brown boxed* positive controls of murine IgGs; *Green boxed* samples from control donors; *Yellow boxed* PM samples; *Red boxed* DM samples; *Blue boxed* sIBM samples. Below are shown representative RPPAs processed with other antibodies. **b** The plot illustrates the linear correlation that exists between the fluorescence intensity (arbitrary units, a.u.) and the amount of PKM2 in HCT116 cell lysates. Protein content in the biopsies was calculated according to the fluorescence intensity obtained in the standard curve of HCT116 cells. For other details see Additional file 1: Figure S1

**Fig. 3:**
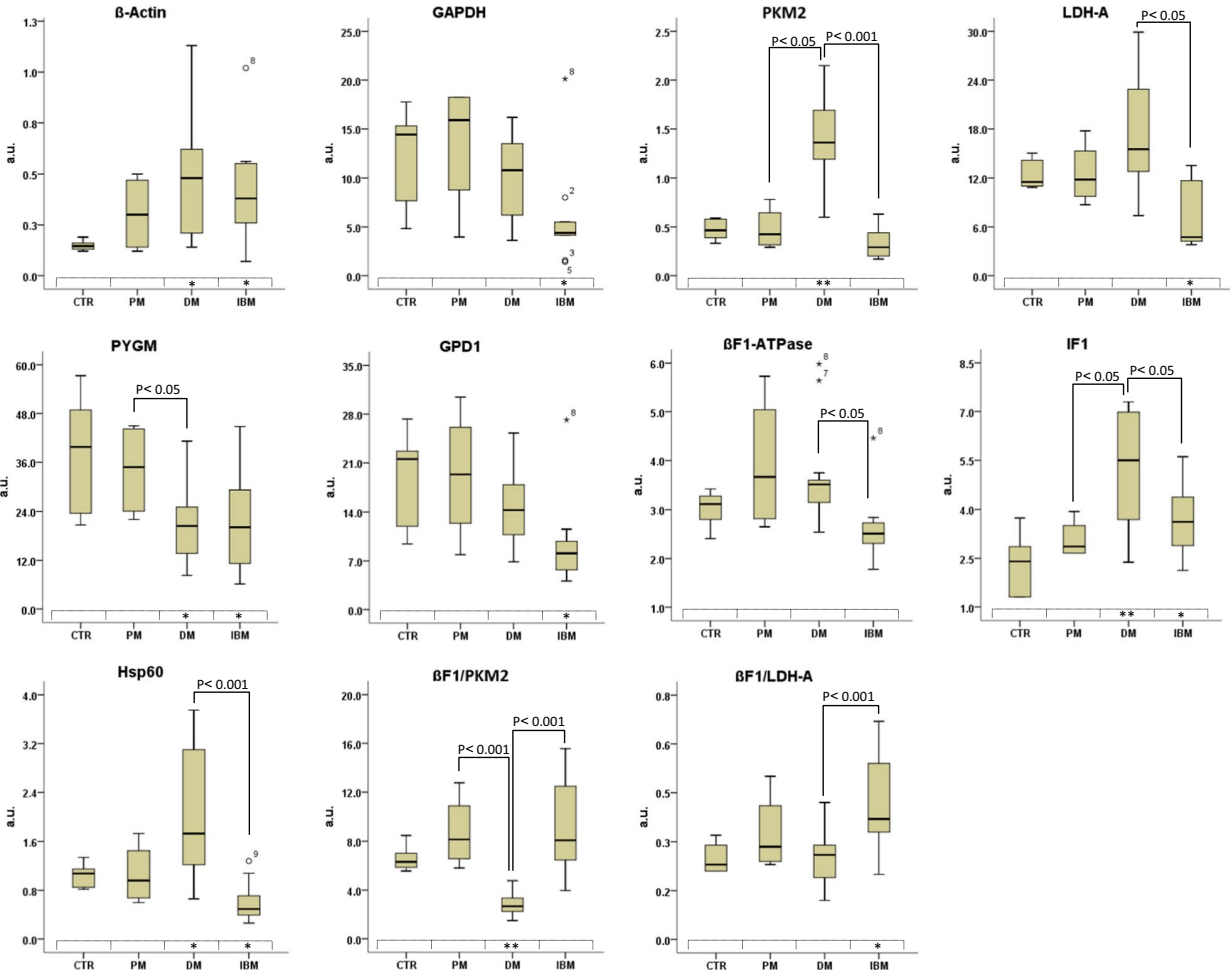
Expression of proteins of energy metabolism in IMs. The *Y axis* indicates the values of intensity (a.u) calculated by interpolation in the linear plot of HCT116 cells. The *X axis* represents patient samples from PM, DM and sIBM and the control (CTR) group. *Box plots* represent the lowest, lower quartile, median, upper quartile and highest observations of each marker in the different groups of pathologies. ○ and *, outlier and extreme values, respectively. The boxed * and ** in the *X*-*axis* indicates a p < 0.05 and p < 0.001 when compared to controls by Student’s t test, respectively

A representative protein microarray illustrating the printing protocol of human muscle biopsies developed with antibodies against the glycolytic PKM2 is shown in Fig. 2a. Microarrays developed with the other antibodies are shown below (Fig. 2a). Protein extracts from muscle biopsies of control (green boxed in Fig. 2a), PM, DM and IBM (yellow, red and blue boxed in Fig. 2a, respectively) were prepared and spotted onto RPPA in triplicate from left to right (Fig. 2a). Increasing amounts of BSA and murine IgGs (black and brown boxed in Fig. 2a, respectively) were spotted in the array as control of the background of the assay and positive control of antibody recognition, respectively. Increasing protein amounts of cellular extracts derived from HCT116 cells were also spotted (magenta boxed in Fig. 2a). The HCT116 extracts revealed a linear increase in fluorescent intensity as the amount of protein increases (Fig. 2b), providing the linear plot of the assay (Fig. 2b, see also Additional file 1: Figure S1 for the rest of the mAbs tested). The arrays illustrate the specific recognition of the corresponding antigen in minute amounts of protein of HCT116 extracts as well as in the biopsies (Fig. 2a). As expected BSA did not show any fluorescent signal, confirming the absence of non-specific binding of the primary and secondary antibodies onto the spotted proteins (Fig. 2a). In contrast, a linear increase in signal was observed in murine IgGs (Fig. 2a), confirming that the secondary antibody works properly. The quantification of the expression of each marker in control (n = 6) and patient (n = 26) biopsies was calculated by interpolating the fluorescent intensity signal obtained in the sample in the standard curve of HCT116 cells (Fig. 2b and Additional file 1: Figure S1). The box plots in Fig. 3 display the results of protein expression in PM, DM and sIBM when compared to controls revealing the inter-individual variation within each group.

**Fig. 4:**
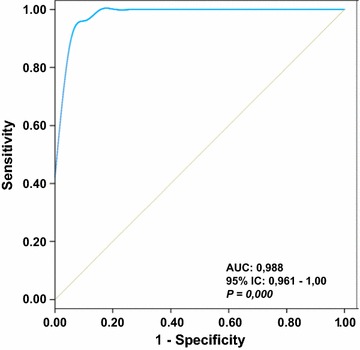
Diagnostic performance of PKM2 in inflammatory myopathies. ROC was plotted to describe PKM2 performance characteristics in a 32 subject cohort. 95% IC 0.961–1.000; P = 0.000 AUC, area under the curve

Interestingly, only DM and sIBM patients showed significant alterations of the expression level of the studied markers when compared to controls (Fig. 3). Muscle biopsies from patients affected with DM showed an increase in the expression of Hsp60 and β-actin concurrent with a similar increase in the expression of PKM2 and the mitochondrial ATPase inhibitor factor IF1 (Fig. 3). These changes occurred in the absence of relevant changes for the expression of other markers and with a significant reduction in PYGM expression (Fig. 3). In contrast, biopsies from sIBM patients showed a significant reduction in the expression of the cytoplasmic GAPDH, LDH-A, PYGM, GPD1 and mitochondrial Hsp60 (Fig. 3). Concurrently, a significant increase in β-actin and IF1 expression (Fig. 3) was observed in sIBM. Overall, and from the point of view of a potential biomarker to distinguish between DM from normal biopsies and the rest of the other IMs stands the sharp increase in PKM2, IF1 and Hsp60 expression (Fig. 3). In fact, PKM2 alone achieved a sensitivity of 96.1% and specificity of 100% (AUC of 0.988) (Fig. 4). Details of the sensitivity (ROC) for IF1 and Hsp60 are provided in Additional file 2: Table S1). In the same line, the down-regulation of glycolytic markers distinguishes sIBM from control biopsies and other IMs (Fig. 3; and see Additional file 2: Table S1).

**Fig. 5:**
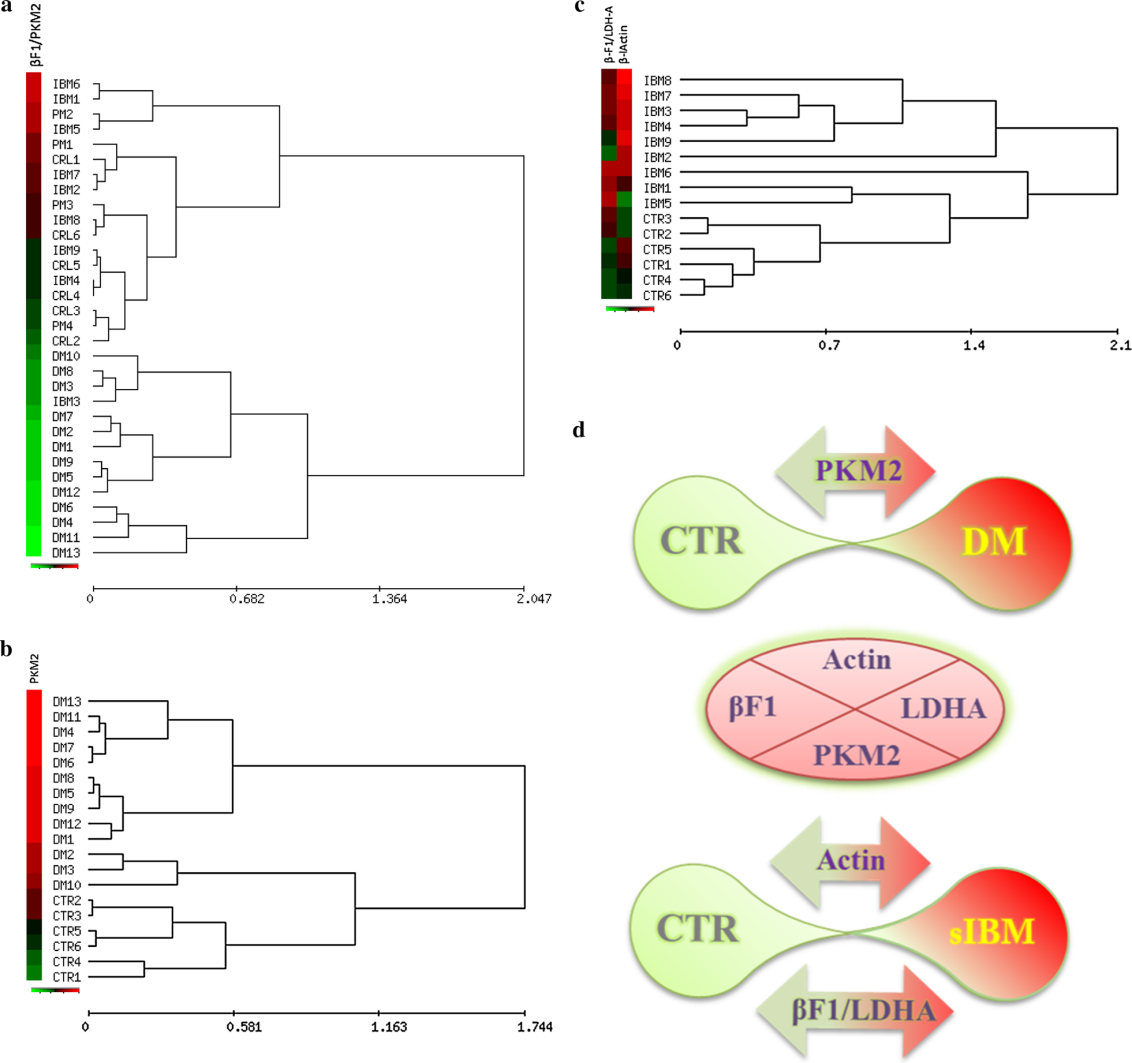
Hierarchical clustering analyses of the biopsies using the expression level of enzymes of energy metabolism. *Rows* indicate type of sample; columns, proteins and derived ratios. Protein expression scores are shown normalized to the mean relative expression level in normal samples in (**a**–**c**), according to a color scale (*below panels*): *red*, high; *black*, normal; *green*, low expression. The dendogram (to the *right* of the matrix) represents overall similarities in expression profiles. The maximum and minimum values of the markers for each cluster are shown. **a** Clustering of control (CTR), DM, PM and sIBM biopsies using βF1-ATPase/PKM2 ratio as biomarker. **b** Clustering of CTR and DM biopsies using PKM2 expression as biomarker. **c** Clustering of CTR and sIBM biopsies using βF1-ATPase/LDHA ratio and β-actin as biomarkers. **d** Scheme showing the contribution of four biomarkers to discriminate CTR, DM and sIBM biopsies attending to high (*red*) or low (*green*) expression levels. For additional details see Table 1

Representative western blot analysis of the three glycolytic markers investigated in RPPAs (Additional file 3: Figure S2) confirmed the higher expression of PKM2 in DM samples and the downregulation of both GAPDH and LDH-A in sIBM biopsies when compared to control or PM samples. Interestingly, PKM2 expression in DM was as high as in the HCT116 carcinoma cell line (Additional file 3: Figure S2). A helpful biomarker that informs of the relative activity of energy provision pathways during development, differentiation and in cancer is the bioenergetic signature [10, 14, 15]. The bioenergetic signature is calculated by the ratio between the catalytic subunit of the H^+^-ATP synthase (β-F1-ATPase) relative to the expression of a glycolytic enzyme [14]. Remarkably, the β-F1-ATPase/PKM2 ratio was significantly diminished in DM providing an excellent bioenergetic marker in order to discriminate this disorder from controls or any other IM (Fig. 5a; Table 1). Likewise, unsupervised hierarchical clustering of the biopsies using the expression of 1, 2 or 3 proteins for aggregation further illustrated the potential of metabolic biomarkers to discriminate normal biopsies from DM (Fig. 5b; Table 1) and from sIBM (Fig. 5c; Table 1) with very high sensitivity and specificity (Fig. 5; Table 1).

**Table 1:** Diagnostic sensitivity of some biomarkers in inflammatory myopathies

Diseases	Markers			Sensitivity (%)	Specificity (%)
*Controls*					
All	β-actin	PKM2	βF1/Hsp60	96	100
DM	PKM2			100	100
sIBM	β-actin	βF1/LDHA		100	100
*DM*					
All	βF1/PKM2			95	100
CTR				100	100
PM				100	100
sIBM				90	100

The table summarizes the power to discriminate (sensitivity) the myopathies by the value of the indicated markers. Sensitivity was calculated accordingly to the classification rate of the positive samples following the formula: Sensitivity = true positives/(true positives + false positives). Specificity = true negatives/(true negatives + false positives)

To further explore the potential bed-side translation of PKM2 as a non-invasive biomarker of DM we investigated whether the inflammation and tissue remodeling that accompanies the disease could be reflected on the plasma levels of PKM2. To this aim we studied the expression of PKM2 in plasma samples of control and IM patients by ELISA and by a modified RPPA technique. Unfortunately, the results obtained revealed that IMs do not modify plasma PKM2 levels to a significant extent when compared to controls (Additional file 4: Figure S3).

**Fig. 6:**
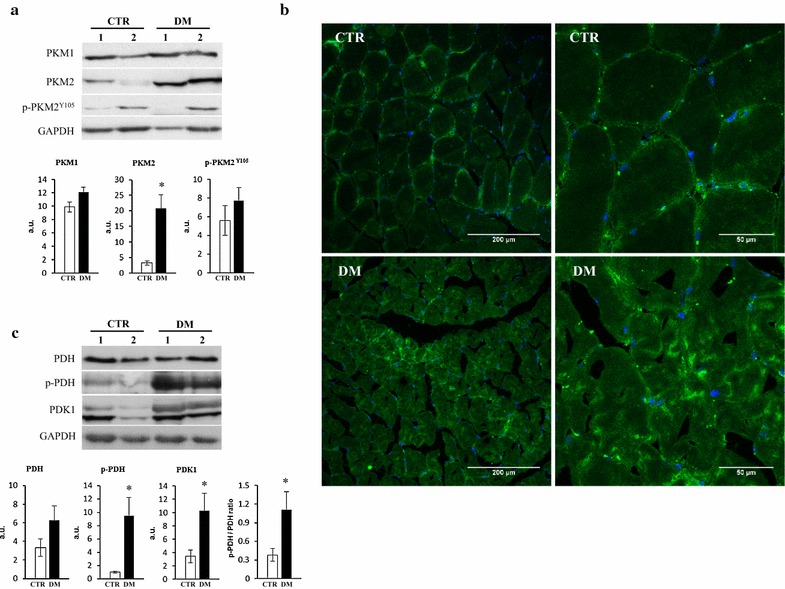
Expression and subcellular localization of PKM2 in muscle of DM patients: impact on pyruvate oxidation. **a** Tissue extracts (20 µg) derived from muscle biopsies of control (CTR) and dermatomyositis (DM) donors were fractionated on SDS-PAGE and blotted against anti-PKM1, anti-PKM2, anti-phospho PKM2^Y105^ and anti-GAPDH. Representative blots of two samples (1,2) per condition are shown. Histograms show the expression of the proteins in arbitrary units (a.u.) ±S.E.M. of five different muscle samples. *p < 0.05 when compared to controls by Student’s t test. **b** Representative confocal images of control (CTR) and dermatomyositis (DM) cryostat muscle sections incubated with anti-PKM2 (*green*) and DAPI (*blue*). Images were taken at 20× and 60×. **c** Same as in **a** but blotted with anti-PDH, anti-phospho PDH, anti-PDK1 and anti-GAPDH. Representative blots of two samples (1,2) per condition are shown. **b** Histograms show the expression of the proteins in arbitrary units (a.u.) ±S.E.M. of five different muscle samples. *p < 0.05 when compared to controls by Student’s t test

In an effort to provide some basic information regarding the expression and regulation of PKM2 in muscle of DM patients we also studied the expression of the PKM1 isoform. We found that expression of PKM1 is not altered in the muscle of DM patients when compared to control samples (Fig. 6a). Due to the very large induction of PKM2 observed in muscle of DM patients (Figs. 3, 6a) the results support that rather than a switch in the expression of PK isoforms DM triggers the specific induction of PKM2. Likewise, analysis of the phosphorylation status of PKM2 in muscle of control and DM patients using the 105Tyr PKM2 antibody revealed, despite the large intra-group variability noted in the phosphorylation of the protein (Fig. 6a), no significant differences between the two groups of samples (Fig. 6a). This finding suggests that PKM2 induction in the muscle of DM patients accounts largely for the active form of the enzyme. Moreover, analysis of the subcellular localization of PKM2 in cryo-sections of muscle samples from control and DM patients by immunofluorescence microscopy revealed that the immunostaining was found in the cytoplasm preferentially clustered towards the sarcolemma and perinuclear region of the cells. We found no evidence for PKM2 translocation into the nucleus of both control and DM muscle cells (Fig. 6b).

To evaluate the potential activation of glycolysis in the muscle of DM patients as a result of the potential limitation of the oxidation of pyruvate in mitochondria we studied the expression of pyruvate dehydrogenase E1α (PDH) and of its less active phosphorylated form (p-PDH) in samples of control and DM patients (Fig. 6c). The results obtained support that glucose oxidation might be partially restricted in the muscle of DM patients when compared to controls because there is a sharp and significant increase in the expression of the less active p-PDH (Fig. 6c) in the absence of relevant changes in total PDH (Fig. 6c). Moreover, these changes were further supported by the parallel and significant concurrent increase in the expression of pyruvate dehydrogenase kinase (PDK1) in muscle samples of DM patients (Fig. 6c).

## Discussion

Diagnosis of IMs relies on histopathologic and immunopathologic examination of muscle biopsies by an experienced laboratory [2, 16]. Specific myositis-related autoantibodies may help diagnosis [17, 18]. Although the diagnosis of DM usually is not difficult based on clinical signs and symptoms, this is not the case with respect to PM and sIBM. The diagnosis of PM is often assessed by exclusion criteria [1], and in turn, usually more than one or two biopsies over time are required before reaching the diagnosis of sIBM. RPPA is a widely adopted high-throughput technique that allows quantitative protein profiling of cells or tissues that is especially indicated for the identification of biomarkers of diagnosis, prognosis and therapeutic response [6, 10, 19, 20]. In fact, RPPA have been recently approved for implementation in clinical trials [6]. Herein, we have interrogated a cohort of muscle biopsies from inflammatory myopathic patients using RPPA and nine validated mAbs against key proteins of energy metabolism to uncover new biomarkers that could help differential diagnosis of IMs. We report that the metabolic biomarkers studied are unable to distinguish control from PM biopsies suggesting the lack of a specific molecular fingerprint of the disease in agreement with its ambiguous clinical profile. In contrast, both DM and sIBM reveal significant differences in the expression of proteins of energy metabolism when compared to controls according to a well-defined clinical profile. Although age, type of muscle fibers and extent of cellular damage in the biopsies are likely to affect protein expression, the low dispersion of the values obtained for each marker suggests that these are not main factors contributing to the differences reported.

Consistent with previous reports [16] we observed an increased expression in β-actin in both DM and sIBM. β-actin overexpression in IMs is the likely consequence of muscle fiber regeneration, a criteria for the classification of IMs [16] also supported by their association with connective tissue disorders [21, 22]. Contrary to the increased expression of β-actin, sIBM biopsies revealed a diminished expression of the glycolytic markers GAPDH and LDHA when compared to controls. Consistently, the expression of GPD1, which forms part of the mitochondrial shuttle of the glycolytic generated NADH, was also diminished in sIBM. Moreover, the expression of PYGM, which is upstream of glycolysis, was also diminished in sIBM biopsies. These findings suggest that muscle fibers of sIBM experience a partial repression of glycolytic metabolism when compared to controls in agreement with recent proteomic findings in this regard [23].

In contrast to the findings in muscle of sIBM, the biopsies of DM patients showed an enhanced expression of the mitochondrial protein Hsp60 and IF1 and a very large increase in the glycolytic PKM2. The βF1-ATPase/LDHA ratio, which has been recently described as a potential biomarker of neuromuscular diseases [13], failed in the discrimination of IMs except for sIBM patients, which showed an increase of the ratio. However, the βF1-ATPase/PKM2 ratio offered a reliable indicator to discriminate DM from any other IM or from control biopsies. In fact, the power of PKM2 as a biomarker in IMs is well demonstrated by the high specificity and sensitivity in the stratification of patients affected with DM when compared to controls or any other group of patients affected with IMs. Overall, we support that the increased expression of PKM2 in DM confers to this protein a great value as biomarker for this particular type of IM, a finding that might benefit some patients by sparing an additional muscle biopsy [16]. Unfortunately, we found that PKM2 levels in plasma of DM patients do not provide a non-invasive biomarker of the disease.

**Fig. 7:**
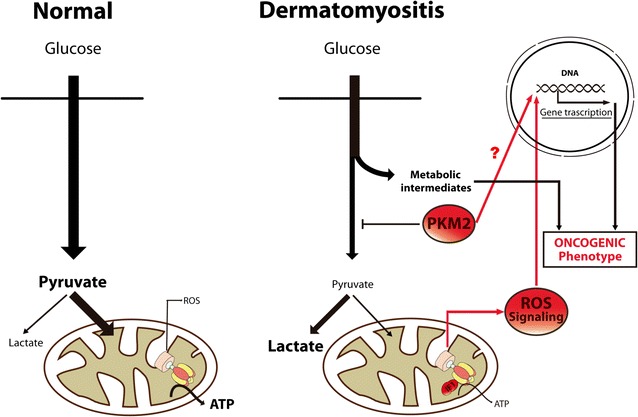
Metabolic reprograming in dermatomyositis. The scheme highlights the primary flux of energy provision pathways in the muscle of normal and DM patients. In normal muscle, most of the glucose taken up by the cell is oxidized in mitochondria to generate a high yield of ATP by oxidative phosphorylation. Mitochondrial activity also generates a little amount of reactive oxygen species (ROS) in the respiratory chain. In contrast, in muscle cells of DM patients glucose is partially oxidized in the cytoplasm to generate metabolic intermediates by the blockage imposed on the glycolytic pathway by the overexpression of the less active PKM2 isoform. Moreover, the overexpression of IF1 in mitochondria also limits cellular ATP availability enforcing aerobic glycolysis and enhancing the production of reactive oxygen species (ROS). Diversion of the glycolytic flux by blockade of glycolysis and oxidative phosphorylation provides the metabolic intermediates that become precursors for the biosynthesis of the macromolecules required for cellular proliferation. In addition, PKM2 can translocate into the nucleus and activates the transcription of several genes involved in cancer development [34–37]. Likewise, the IF1-mediated inhibition of the H^+^-ATP synthase generates a ROS signal that activates in the nucleus programs involved in proliferation, cell death resistance and invasion [38–44]. Thus, the concurrent increase of PKM2 and IF1 expression in the muscle of DM patients leads to metabolic rewiring and ROS signaling that are hallmarks of the oncogenic phenotype

It is generally agreed that cancer is more frequently associated with DM than with PM [2] and a clear increase of cancer related mortality has been reported in DM patients when compared to PM patients [1, 24–26]. A distinctive feature of cancer cells is the enhanced aerobic glycolysis [14, 27]. Pyruvate kinase (PK) is the enzyme that catalyzes the final step in glycolysis, converting phosphoenolpyruvate (PEP) to pyruvate [28]. Four different isoforms of pyruvate kinase exist: type-R and type-L are generated by alternative splicing of the *PKLR* gene and are expressed in erythrocytes and in liver, respectively [29]. PKM1 is the isoform expressed in adult skeletal muscle while PKM2, which results from alternative splicing of the *PKM* gene is expressed exclusively in embryonic and proliferating tissues. Notably, PKM2 is allosterically regulated due to its possibility to switch from a dimeric low-active form to a tetrameric very high active form [30–32]. In addition, phosphorylation of S37 and Y105 in PKM2 prevents the binding of the PKM2 cofactor fructose-1,6-bisphosphate, thus inhibiting the active tetrameric form of PKM2 which promotes aerobic glycolysis and tumor growth [33] (Fig. 5). Our findings stress the idea that DM triggers an increase in the expression of PKM2 rather than a switch in the expression of muscle isoforms. Likewise, the increased expression of PKM2 in muscle of DM patients is in its non-phosphorylated active state. Moreover, PKM2 also has a “non-metabolic” role in tumorigenesis since its translocation into the nucleus regulates gene transcription of several pathways involved in metabolic reprogramming, cell proliferation and cancer development [34–37]. Although we did not observe any nuclear localization of PKM2 in muscle of DM patients, we cannot exclude this possibility (Fig. 7) because its nuclear translocation might represent a late event in the pro-oncogenic development of the disease.

Strikingly, and highly consistent with the increased cancer incidence observed in DM patients [1, 24–26], we found an elevated expression of IF1 in DM biopsies. In fact, IF1 is highly overexpressed in most prevalent human carcinomas [12, 38]. Overexpression of IF1 interferes with oxidative phosphorylation by inhibiting the H^+^-ATP synthase promoting aerobic glycolysis and signaling, via reactive oxygen species (ROS), a pro-oncogenic phenotype (Fig. 7) that enhances proliferation, invasion and cell survival [12, 39, 40]. More recently, the overexpression of IF1 in human hepatocellular carcinomas, bladder and gastric cancers and in gliomas has provided a valuable biomarker of bad cancer prognosis [41–44]. Mechanistically, the pro-oncogenic features of IF1 over-expression stem from its effects on favoring proliferation, cell death resistance, epithelial mesenchymal transition and angiogenesis [39–41, 45]. Altogether, we suggest that the increased expression of PKM2 and IF1 could cooperate to promote a metabolic phenotype in DM that is prone for the onset of oncogenesis (Fig. 7). Consistent with this idea, our findings support that pyruvate oxidation in muscle of DM patients might be partially restricted when compared to controls by the diminished fraction of active pyruvate dehydrogenase present in mitochondria (Fig. 6c). We suggest that these findings might stimulate the development of future basic investigations and therapeutic approaches for the management of DM patients in order to prevent their higher cancer incidence.

## Conclusions

Our study highlights the usefulness of combining RPPA and proteins of energy metabolism as a tool to identify novel biomarkers in rare myopathic diseases. We further illustrate the clinical relevance of PKM2 and IF1, two proteins that respectively act on glycolysis and oxidative phosphorylation, as novel biomarkers of dermatomyositis and potential metabolic drivers of the higher cancer incidence observed in this inflammatory myopathy.

## Additional files

**Additional file 1: Figure S1.** Linear correlation between the fluorescence intensity and the content of native proteins. HCT116 cell line extracts (0–1 μg/μl) were spotted in the arrays (see Fig. 2a). Significant linear correlations were obtained between the fluorescence intensity (arbitrary units, a.u.) of the spots and the amount of the protein interrogated in the arrays. Protein concentrations in the biopsies were calculated by interpolation in the respective linear plots.

**Additional file 2: Table S1.** Summary of diagnostic sensitivity of metabolic biomarkers in inflammatory myopathies. AUC, Area Under the Curve.

**Additional file 3: Figure S2.** Overexpression of PKM2 in DM. **a** Tissue extracts (30 µg) derived from two randomly selected muscle biopsies of control donors (CTR), polymyositis (PM), dermatomyositis (DM) and sporadic inclusion body myositis (IBM) and of the HCT116 cell line were fractionated on SDS-PAGE and blotted against anti-PKM2, anti-GAPDH and anti-LDH-A. Electrophoretic migration of the protein is indicated to the left of each blot. **b** Histograms show the expression of the proteins (a.u.) when compared to the expression in HCT116 cells.

**Additional file 4: Figure S3.** Plasma levels of PKM2 by ELISA and RPPA. **a** Determination of PKM2 by ELISA in plasma samples of DM, sIBM and CRL patients. **b** Upper panel, scheme of printing of the plasma samples from CRL, sIBM, DM and PM patients. One nl of 1:20 diluted samples were spotted in quadruplicate. Black boxed: negative controls of BSA; Magenta boxed: standard curves of HCT116 cells; Brown boxed: positive controls of murine IgGs; Orange boxed: standard curve of PKM2 recombinant protein; Green boxed: samples from control donors (CTR); Blue boxed: samples from sIBM patients; Red boxed: samples from DM patients; Yellow boxed: sample from PM patients. Lower panel, parallel array processed with goat anti-mouse IgGs CF647 as negative control. Please note the lack of cross-reactivity against the human plasma IgGs. **c** The histogram shows the plasma levels of PKM2 quantified by RPPA assay. The results shown are mean ± S.E.M.

## Abbreviations

β-F1-ATPase: β-subunit of the H^+^-ATP synthase
DM: dermatomyositis
GAPDH: glyceraldehyde-3-phosphate dehydrogenase
GPD1: glycerol-3-phosphate dehydrogenase 1
Hsp60: heat shock protein 60 kDa
IBM: inclusion body myositis
IF1: ATPase Inhibitory Factor 1
IM: inflammatory myopathy
LDH-A: lactate dehydrogenase A
PDH: pyruvate dehydrogenase E1α
p-PDH: phospho-pyruvate dehydrogenase E1α
PDK1: pyruvate dehydrogenase kinase 1
PYGM: glycogen phosphorylase
PK: pyruvate kinase
PKM1: pyruvate kinase M1
PKM2: pyruvate kinase M2
PM: polymyositis
RPPA: reverse phase protein microarray

## References

[1] Dalakas MC, Hohlfeld R (2003). Polymyositis and dermatomyositis. Lancet.

[2] Dalakas MC (2015). Inflammatory muscle diseases. N Engl J Med.

[3] Bohan A, Peter JB (1975). Polymyositis and dermatomyositis (first of two parts). N Engl J Med.

[4] Mastaglia FL, Phillips BA (2002). Idiopathic inflammatory myopathies: epidemiology, classification, and diagnostic criteria. Rheum Dis Clin North Am.

[5] Catalan M, Selva-O’Callaghan A, Grau JM (2014). Diagnosis and classification of sporadic inclusion body myositis (sIBM). Autoimmun Rev.

[6] Mueller C, Liotta LA, Espina V (2010). Reverse phase protein microarrays advance to use in clinical trials. Mol Oncol.

[7] Tibes R, Qiu Y, Lu Y, Hennessy B, Andreeff M, Mills GB (2006). Reverse phase protein array: validation of a novel proteomic technology and utility for analysis of primary leukemia specimens and hematopoietic stem cells. Mol Cancer Ther.

[8] Michaud GA, Salcius M, Zhou F, Bangham R, Bonin J, Guo H (2003). Analyzing antibody specificity with whole proteome microarrays. Nat Biotechnol.

[9] Strausberg RL, Simpson AJ, Old LJ, Riggins GJ (2004). Oncogenomics and the development of new cancer therapies. Nature.

[10] Aldea M, Clofent J, Nunez de Arenas C, Chamorro M, Velasco M, Berrendero JR (2011). Reverse phase protein microarrays quantify and validate the bioenergetic signature as biomarker in colorectal cancer. Cancer Lett.

[11] Acebo P, Giner D, Calvo P, Blanco-Rivero A, Ortega AD, Fernandez PL (2009). Cancer abolishes the tissue type-specific differences in the phenotype of energetic metabolism. Transl Oncol.

[12] Sanchez-Cenizo L, Formentini L, Aldea M, Ortega AD, Garcia-Huerta P, Sanchez-Arago M (2010). Up-regulation of the ATPase Inhibitory Factor 1 (IF1) of the mitochondrial H^+^-ATP synthase in human tumors mediates the metabolic shift of cancer cells to a Warburg phenotype. J Biol Chem.

[13] Santacatterina F, Chamorro M, Nuñez de Arenas C, Navarro C, Martin MA, Cuezva JM (2015). Quantitative analysis of proteins of metabolism by reverse phase protein microarrays identifies potential biomarkers of rare neuromuscular diseases. J Trans Med.

[14] Cuezva JM, Ortega AD, Willers I, Sanchez-Cenizo L, Aldea M, Sanchez-Arago M (2009). The tumor suppressor function of mitochondria: translation into the clinics. Biochim Biophys Acta.

[15] Yizhak K, Le Devedec SE, Rogkoti VM, Baenke F, de Boer VC, Frezza C (2014). A computational study of the Warburg effect identifies metabolic targets inhibiting cancer migration. Mol Syst Biol.

[16] Selva OCA, Trallero AE (2008). Inflammatory myopathies. Dermatomyositis, polymyositis, and inclusion body myositis. Reumatol Clin.

[17] Gunawardena H, Betteridge ZE, McHugh NJ (2009). Myositis-specific autoantibodies: their clinical and pathogenic significance in disease expression. Rheumatology (Oxford).

[18] Benveniste O (2015). Acquired inflammatory myopathies: interest of specific autoantibodies for their classification. Rev Prat.

[19] Ledford H (2014). Metabolic quirks yield tumour hope. Nature.

[20] Sereni MI, Pierobon M, Angioli R, Petricoin EF, Frederick MJ (2013). Reverse phase protein microarrays and their utility in drug development. Methods Mol Biol.

[21] Dalakas MC (1991). Polymyositis, dermatomyositis and inclusion-body myositis. N Engl J Med.

[22] van der Kooi AJ, de Visser M (2014). Idiopathic inflammatory myopathies. Handb Clin Neurol.

[23] Parker KC, Kong SW, Walsh RJ, Salajegheh M, Moghadaszadeh B (2009). Fast-twitch sarcomeric and glycolytic enzyme protein loss in inclusion body myositis. Muscle Nerve.

[24] Sigurgeirsson B, Lindelof B, Edhag O, Allander E (1992). Risk of cancer in patients with dermatomyositis or polymyositis. A population-based study. N Engl J Med.

[25] Hill CL, Zhang Y, Sigurgeirsson B, Pukkala E, Mellemkjaer L, Airio A (2001). Frequency of specific cancer types in dermatomyositis and polymyositis: a population-based study. Lancet.

[26] Chen YJ, Wu CY, Huang YL, Wang CB, Shen JL, Chang YT (2010). Cancer risks of dermatomyositis and polymyositis: a nationwide cohort study in Taiwan. Arthritis Res Ther.

[27] Vander Heiden MG, Cantley LC, Thompson CB (2009). Understanding the Warburg effect: the metabolic requirements of cell proliferation. Science.

[28] Robinson JL, Rose IA (1972). The proton transfer reactions of muscle pyruvate kinase. J Biol Chem.

[29] Tsutsumi H, Tani K, Fujii H, Miwa S (1988). Expression of L- and M-type pyruvate kinase in human tissues. Genomics.

[30] Mazurek S (2011). Pyruvate kinase type M2: a key regulator of the metabolic budget system in tumor cells. Int J Biochem Cell Biol.

[31] Anastasiou D, Yu Y, Israelsen WJ, Jiang JK, Boxer MB, Hong BS (2012). Pyruvate kinase M2 activators promote tetramer formation and suppress tumorigenesis. Nat Chem Biol.

[32] DeLaBarre B, Hurov J, Cianchetta G, Murray S, Dang L (2014). Action at a distance: allostery and the development of drugs to target cancer cell metabolism. Chem Biol.

[33] Hitosugi T, Kang S, Vander Heiden MG, Chung TW, Elf S, Lythgoe K (2009). Tyrosine phosphorylation inhibits PKM2 to promote the Warburg effect and tumor growth. Sci Signal.

[34] Iqbal MA, Gupta V, Gopinath P, Mazurek S, Bamezai RN (2014). Pyruvate kinase M2 and cancer: an updated assessment. FEBS Lett.

[35] Wang HJ, Hsieh YJ, Cheng WC, Lin CP, Lin YS, Yang SF (2014). JMJD5 regulates PKM2 nuclear translocation and reprograms HIF-1alpha-mediated glucose metabolism. Proc Natl Acad Sci USA.

[36] Luo W, Hu H, Chang R, Zhong J, Knabel M, O’Meally R (2011). Pyruvate kinase M2 is a PHD3-stimulated coactivator for hypoxia-inducible factor 1. Cell.

[37] Yang W, Xia Y, Ji H, Zheng Y, Liang J, Huang W (2011). Nuclear PKM2 regulates beta-catenin transactivation upon EGFR activation. Nature.

[38] Sanchez-Arago M, Formentini L, Martinez-Reyes I, Garcia-Bermudez J, Santacatterina F, Sanchez-Cenizo L (2013). Expression, regulation and clinical relevance of the ATPase Inhibitory Factor 1 in human cancers. Oncogenesis.

[39] Formentini L, Sánchez-Aragó M, Sánchez-Cenizo L, Cuezva JM (2012). The mitochondrial ATPase Inhibitory Factor 1 (IF1) triggers a ROS-mediated retrograde pro-survival and proliferative response. Mol Cell.

[40] Santacatterina F, Sanchez-Cenizo L, Formentini L, Mobasher MA, Casas E, Rueda CB (2016). Down-regulation of oxidative phosphorylation in the liver by expression of the ATPase Inhibitory Factor 1 induces a tumor-promoter metabolic state. Oncotarget.

[41] Song R, Song H, Liang Y, Yin D, Zhang H, Zheng T (2014). Reciprocal activation between ATPase Inhibitory Factor 1 and NF-kappaB drives hepatocellular carcinoma angiogenesis and metastasis. Hepatology.

[42] Wei S, Fukuhara H, Kawada C, Kurabayashi A, Furihata M, Ogura S (2015). Silencing of ATPase Inhibitory Factor 1 inhibits cell growth via cell cycle arrest in bladder cancer. Pathobiology.

[43] Yin T, Lu L, Xiong Z, Wei S, Cui D (2015). ATPase Inhibitory Factor 1 is a prognostic marker and contributes to proliferation and invasion of human gastric cancer cells. Biomed Pharmacother.

[44] Wu J, Shan Q, Li P, Wu Y, Xie J, Wang X (2015). ATPase Inhibitory Factor 1 is a potential prognostic marker for the migration and invasion of glioma. Oncol Lett.

[45] Garcia-Bermudez J, Cuezva JM (2016). The ATPase Inhibitory Factor 1 (IF1): a master regulator of energy metabolism and of cell survival. Biochim Biophys Acta.

