# Supplementary figures and images for: Pyruvate kinase M2 and the mitochondrial ATPase Inhibitory Factor 1 provide novel biomarkers of dermatomyositis: a metabolic link to oncogenesis
Source: J Transl Med. 2017 Feb 10;15:29. doi: 10.1186/s12967-017-1136-5 (PMC5301421; doi:10.1186/s12967-017-1136-5)

## Slide 1
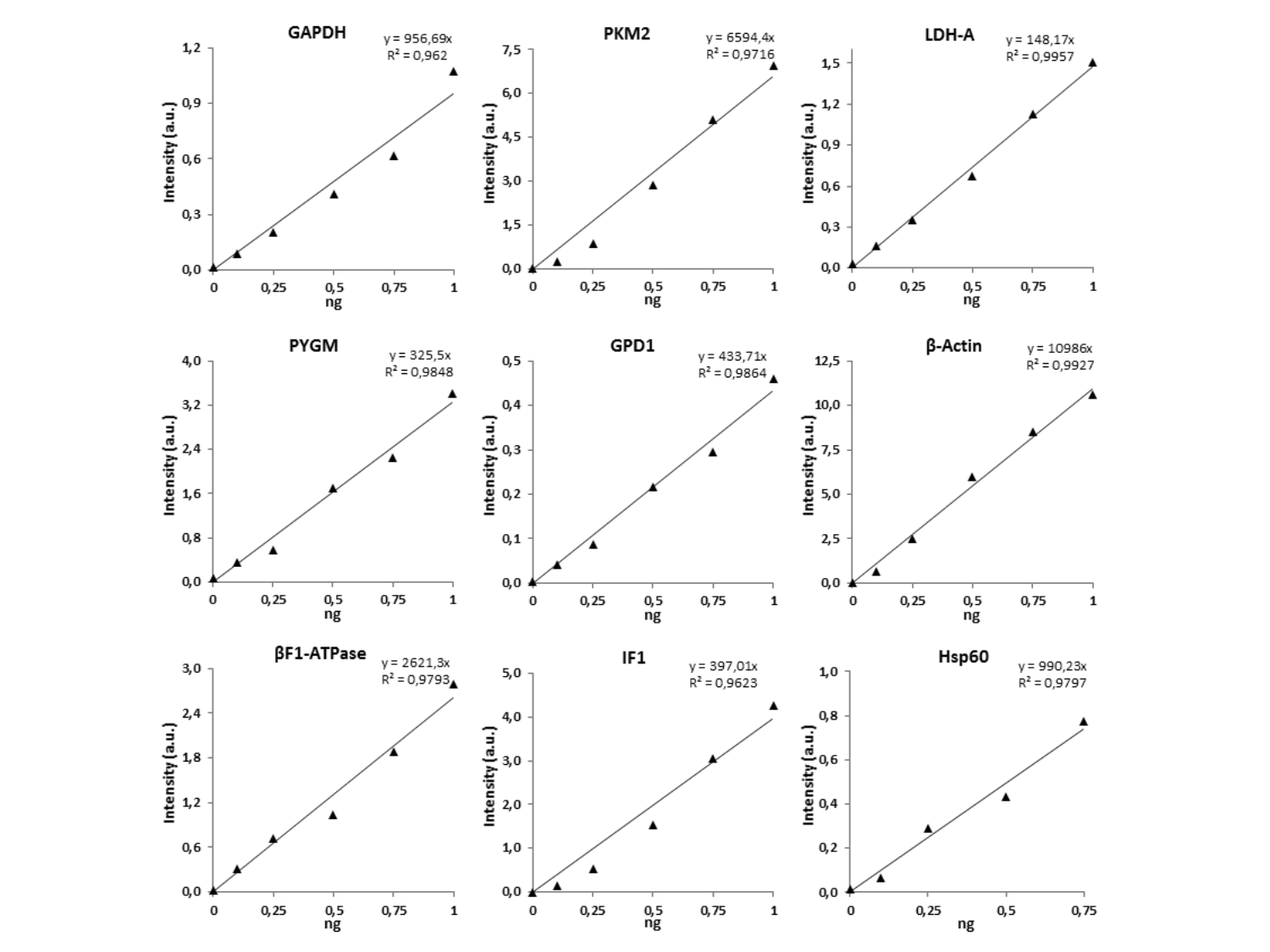

Supplement: Supplementary file 1 — Additional file 1: Figure S1. Linear correlation between the fluorescence intensity and the content of native proteins. HCT116 cell line extracts (0–1 μg/μl) were spotted in the arrays (see Fig. 2a). Significant linear correlations were obtained between the fluorescence intensity (arbitrary units, a.u.) of the spots and the amount of the protein interrogated in the arrays. Protein concentrations in the biopsies were calculated by interpolation in the respective linear plots. [file 12967_2017_1136_MOESM1_ESM.pptx]

## Slide 1
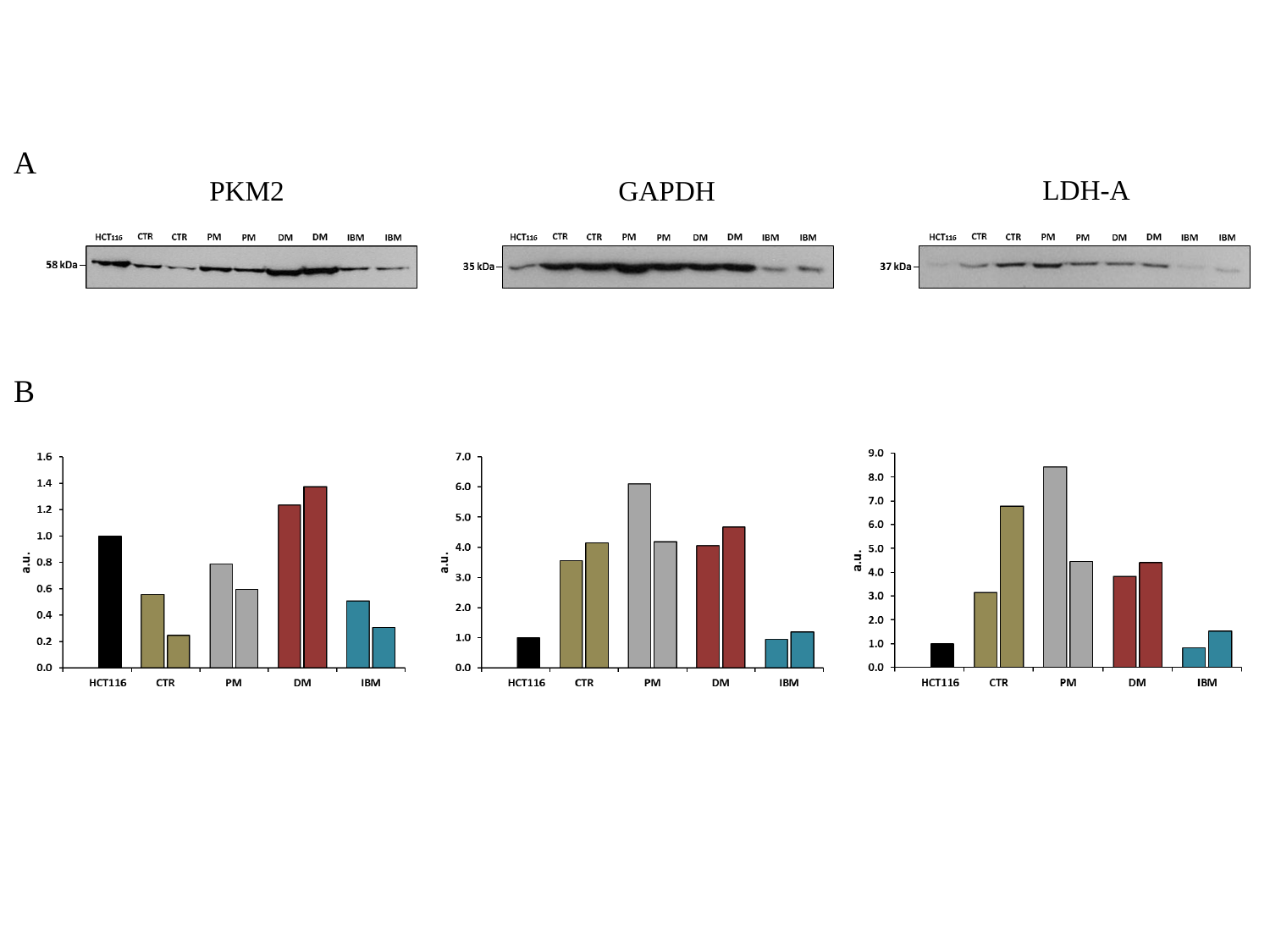

A
LDH-A
PKM2
GAPDH
B

Supplement: Supplementary file 3 — Additional file 3: Figure S2. Overexpression of PKM2 in DM. a Tissue extracts (30 µg) derived from two randomly selected muscle biopsies of control donors (CTR), polymyositis (PM), dermatomyositis (DM) and sporadic inclusion body myositis (IBM) and of the HCT116 cell line were fractionated on SDS-PAGE and blotted against anti-PKM2, anti-GAPDH and anti-LDH-A. Electrophoretic migration of the protein is indicated to the left of each blot. b Histograms show the expression of the proteins (a.u.) when compared to the expression in HCT116 cells. [file 12967_2017_1136_MOESM3_ESM.pptx]
