# Supplementary material for: Pyruvate kinase M2 and the mitochondrial ATPase Inhibitory Factor 1 provide novel biomarkers of dermatomyositis: a metabolic link to oncogenesis
Source: J Transl Med. 2017 Feb 10;15:29. doi: 10.1186/s12967-017-1136-5 (PMC5301421; doi:10.1186/s12967-017-1136-5)
Supplement: Supplementary file 2 — Additional file 2: Table S1. Summary of diagnostic sensitivity of metabolic biomarkers in inflammatory myopathies. AUC, Area Under the Curve. [file 12967_2017_1136_MOESM2_ESM.docx]

**Additional file 2.**

| **IMs** | **Biomarker** | **AUC** | **95 % CI** | ***P (Area 0.5)*** |
| --- | --- | --- | --- | --- |
| **DM** | PKM2 | 0.988 | 0.961 – 1.00 | *<0.000* |
|  | IF1 | 0.826 | 0.669 – 0.983 | *<0.002* |
|  | Hsp60 | 0.844 | 0.697 – 0.991 | *<0.001* |
| **sIBM** | GAPDH | 0.763 | 0.552 – 0.974 | *<0.022* |
|  | LDH-A | 0.860 | 0.709 – 1.00 | *<0.002* |
|  | PYGM | 0.628 | 0.404 – 0.852 | *<0.267* |
|  | GPD1 | 0.807 | 0.612 – 1.00 | *<0.008* |
|  | Hsp60 | 0.882 | 0.747 – 1.00 | *<0.001* |

**Summary of diagnostic sensitivity of metabolic biomarkers in inflammatory myopathies.** AUC, Area under the ROC curve
