# Supplementary material for: Pyruvate kinase M2 and the mitochondrial ATPase Inhibitory Factor 1 provide novel biomarkers of dermatomyositis: a metabolic link to oncogenesis
Source: J Transl Med. 2017 Feb 10;15:29. doi: 10.1186/s12967-017-1136-5 (PMC5301421; doi:10.1186/s12967-017-1136-5)
Supplement: Supplementary file 4 — Additional file 4: Figure S3. Plasma levels of PKM2 by ELISA and RPPA. a Determination of PKM2 by ELISA in plasma samples of DM, sIBM and CRL patients. b Upper panel, scheme of printing of the plasma samples from CRL, sIBM, DM and PM patients. One nl of 1:20 diluted samples were spotted in quadruplicate. Black boxed: negative controls of BSA; Magenta boxed: standard curves of HCT116 cells; Brown boxed: positive controls of murine IgGs; Orange boxed: standard curve of PKM2 recombinant protein; Green boxed: samples from control donors (CTR); Blue boxed: samples from sIBM patients; Red boxed: samples from DM patients; Yellow boxed: sample from PM patients. Lower panel, parallel array processed with goat anti-mouse IgGs CF647 as negative control. Please note the lack of cross-reactivity against the human plasma IgGs. c The histogram shows the plasma levels of PKM2 quantified by RPPA assay. The results shown are mean ± S.E.M. [file 12967_2017_1136_MOESM4_ESM.pptx]

## Slide 1
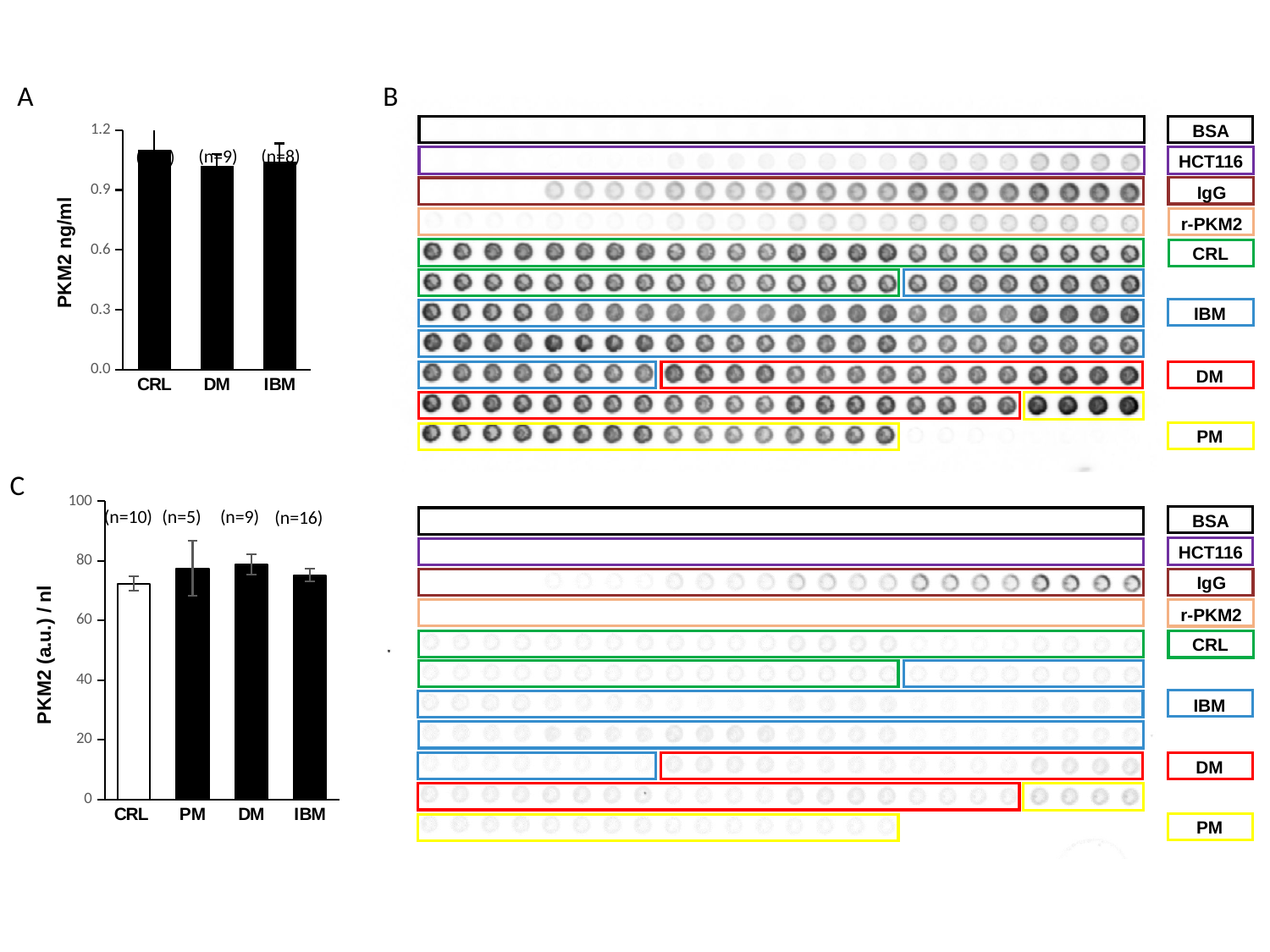

A
B
### Chart
| Category | |
|---|---|
| CRL | 1.097101186555913 |
| DM | 1.018690029748956 |
| IBM | 1.0399710266836635 |
BSA
(n=9)
(n=8)
(n=8)
HCT116
IgG
r-PKM2
CRL
IBM
DM
PM
C
### Chart
| Category | |
|---|---|
| CRL | 72.4189535309184 |
| PM | 77.48481522136845 |
| DM | 78.84213521974225 |
| IBM | 75.20581778265641 |
(n=5)
(n=9)
(n=10)
(n=16)
BSA
HCT116
IgG
r-PKM2
CRL
IBM
DM
PM
